# Metaheuristic-based optimal energy assessment of hybrid multi-effect evaporator with synergy of solar and wind energy sources

**DOI:** 10.1016/j.heliyon.2025.e41653

**Published:** 2025-01-07

**Authors:** Smitarani Pati, Nandan Kumar Navin, Om Prakash Verma, Dwesh Kumar Singh, Tarun Kumar Sharma, Saurabh Agarwal, Santos Gracia Villar, Luis Alonso Dzul Lopez, Imran Ashraf

**Affiliations:** a Department of Electrical Engineering, Dr B R Ambedkar National Institute of Technology Jalandhar, Punjab 144011, India; b Department of Instrumentation and Control Engineering, Dr B R Ambedkar National Institute of Technology Jalandhar, Punjab 144011, India; c Department of Mechanical Engineering, Dr B R Ambedkar National Institute of Technology Jalandhar, Punjab 144011, India; d Department of CSE, Shobhit University, Gangoh, India; e Information and Communication Engineering, Yeungnam University, Gyeongsan 38541, Korea; f Universidad Europea del Atlántico, Isabel Torres 21, 39011 Santander, Spain; g Universidad Internacional Iberoamericana, Campeche 24560, Mexico; h Universidade Internacional do Cuanza, Cuito, Bié, Angola; i Universidad de La Romana, La Romana, Dominican Republic

**Keywords:** Multi-effect evaporator, Energy saving schemes, Energy efficiency analysis, Nonlinear constraint optimization, Renewable thermal assistance

## Abstract

This study emphasizes a multi-pronged approach to improving the energy efficiency of Multi-Effect Evaporator (MEE) in the paper industry. By incorporating traditional Energy-Saving Schemes (ESSs) and innovative renewable energy sources, the study demonstrates significant potential for reducing energy consumption and environmental impact, making it a decisive pathway for industrial sustainability. Key ESS strategies include Thermo-Vapor Compressors, Feed Preheaters, and Steam- and Feed-Split, which are employed to enhance Steam Economy (SE) to evaluate MEE efficiency. This integration results in a 67.93% enhancement in SE, reducing energy consumption significantly. Further, SE enhancement is achieved by integrating flash tanks that capture and reuse excess heat, which boosts SE by an additional 5.89%, leading to a total improvement of 73% without additional energy consumption. A significant innovation in the study is the integration of Linear Fresnel Reflectors (LFRs) based solar collectors and turbine-based wind energy sources to power the MEE and reduce reliance on conventional energy. This hybrid system decreases energy dependence by 62% for the base MEE and 34% for the hybrid MEE. The results are validated by comparing them with existing studies, confirming the effectiveness of the proposed method and offering significant energy and environment savings.

## 1 Introduction

Black liquor (BL) is a biomass source extracted from the digester as industrial waste during the paper-making process, typically containing 15% to 20% solids [1]. It comprises chemicals used in pulp cleaning, along with a significant percentage of lignin, hemicellulose, and 80% to 85% of water [2]. Annually, the paper industry produces approximately 170 million tons of BL, capable of generating about 2EJ of energy [2], making it a promising resource for energy generation [3]. Multi-effect evaporators (MEE) are crucial in concentrating BL for use as biofuel [4]. MEEs, consisting of multiple stages, utilize the latent heat of secondary steam to concentrate BL, but they also constitute a significant energy-consuming section of the paper industry, consuming over 30% of total industrial energy [5]. Consequently, various efforts have been made to integrate energy-saving schemes into MEE operations. This study focuses on a backward feed falling film seven-effect MEE located at a paper mill in Saharanpur, Utter Pradesh (UP), India, to explore renewable energy implementations and reduce energy consumption [6].

In recent years, various energy-saving schemes (ESSs) have been proposed to improve the energy efficiency of MEEs. These include feed preheaters [7], thermo-vapor compression (TVC) [8], [9], [10], mechanical vapor compression [11], and flash tanks [12], [13]. Further, modifications to operations including feed- and steam-splitting have also been suggested to reduce energy consumption [6], [14]. Hybrid models combining these ESSs have shown further improvements in energy efficiency [14], [15], [16]. Hence, this study aims to analyze process parameters to optimize Steam Economy (SE) in two separate MEE configurations, comparing base (b-) and hybrid (h-) models. SE is defined as the ratio of the difference between input and output black liquor to the amount of steam utilized to operate the system, which is termed Steam Consumption (SC), which is also a prime objective entity that needs to be minimized. The hybrid model incorporates TVC, feed preheater, flash tanks, and steam- and feed-splitting.

Integrating ESSs with MEEs offers limited energy savings. However, incorporating renewable energy can lead to self-sustainability, significantly reducing reliance on conventional energy sources. While the integration of renewables with MEEs in the paper industry remains largely unexplored, it's a concept ripe for innovation. Unlike the desalination industry, where renewables like solar and wind are commonly integrated with MEEs, there's a lack of literature on hybridized renewables in the paper sector [9], [17], [18]. Nonetheless, simulation-based studies have explored the potential of solar collectors in conjunction with MEEs [19], [20].

While solar energy integration is crucial in many industries, its availability is limited to daytime without storage [21]. Wind energy, on the other hand, offers continuous power, making it a competitive renewable source. Hybrid integration of solar and wind energy is proposed to replace conventional energy usage in operating MEEs. Although commonly used in desalination plants, this approach is novel for other industries like papermaking due to differing industrial locations. Among solar alternatives, concentrating solar power (CSP) plants [22], particularly linear Fresnel reflectors (LFR), are promising for the paper industry due to their ability to produce direct steam at low temperatures, reducing traditional energy demands for MEEs. Thus, this investigation considers integrating LFR solar fields and wind energy sources for enhanced energy efficiency.

Analyzing the energy efficiency of ESSs integrated with MEEs involves developing steady-state models based on the first law of thermodynamics. These models incorporate mass and thermal balances along with heat transfer rate correlations for each MEE effect. Due to varying process variables in each effect, nonlinear behavior is observed. Solving for unknown variables such as temperature (Ti), Black Liquor (BL) flow rate (Li), and steam consumption (V0) is typically done using iterative techniques like the Newton-Raphson method [23], [24], [25]. However, this approach can be computationally complex due to the formation of the Jacobian matrix and is also sensitive to initial guesses. Other methods such as pinch analysis [26], [27], [28] and interior-point methods (IPMs) [14] offer alternatives but require further refinement, especially with increased MEE effects. Advancements in technology have led to the implementation of optimization approaches like nonlinear programming and metaheuristic techniques such as genetic algorithm (GA) [29], differential evolution (DE), particle swarm optimization (PSO) [30], WCA [31], ABCA [32], [33], FDA [34] and AEFA [35], SCA [19] to achieve robust solutions efficiently. Furthermore, there is also a need to verify the efficiency and robustness of newly developed metaheuristic algorithms employed to solve these computationally complex nonlinear energy models.

Hence, a newly developed nature-inspired algorithm named the Walrus optimization algorithm (WaOA) inspired by the hunting behavior of walruses is proposed to search for the optimum process parameters [36]. Energy calculations for solar and wind sources are based on collected weather data for the region. Developing a simulation-based design for MEEs, incorporating solar and wind energy, is essential to estimate generating capacity and determine installation locations. This study addresses energy-related challenges in MEEs and explores energy management schemes to enhance BL concentration efficiently. The prime objectives of the present instigation are

• Formulate an optimization problem to maximize steam economy (SE) within a constrained environment.
• Employ the WaOA to identify the best optimal values.
• Reutilize waste heat through flash tanks.
• Incorporate LFR solar fields and wind energy to offset thermal demand.
• Compare and validate models integrating ESSs with MEEs.

Further, related work is discussed in Section 2 while the system description is provided in Section 3. Section 4 introduces the proposed approach. Renewable energy integration is discussed in Section 5 while wind energy is discussed in Section 6. Section 7 discusses the results and performance comparison with existing works. Section 8 concludes the study.

## 2 Related work

Hybrid renewable energy systems (HRES) combining solar, wind, biomass, and other sources have gained increasing attention due to their potential to provide reliable and sustainable energy. Optimization algorithms are pivotal in enhancing the efficiency and effectiveness of such systems. Various studies have contributed to this growing field by developing advanced algorithms and analyzing the technical and economic performance of HRES under different conditions.

An enhanced Cuckoo Optimization Algorithm (COA) integrating a Gaussian Mixture Model (GMM) was introduced to improve the accuracy and convergence rate of the Optimal Power Flow (OPF) problem in solar and wind hybrid systems [37]. This work complements the efforts of Mishra and Shaik, who applied the African Vulture Optimization Algorithm (AVOA) to balance economic and environmental objectives in diesel-wind-solar microgrids [38]. By minimizing operational costs and emissions, AVOA tackles challenges in sustainable microgrid design, particularly in off-grid and remote locations.

On the environmental front, comprehensive exergy, exergoeconomic, and exergo environmental analysis of a hybrid solar, wind, and marine energy system highlights the importance of considering multiple performance metrics in HRES design [39]. This study emphasizes the thermodynamic efficiency and environmental impact of integrating various renewable sources, reinforcing the global focus on carbon-free energy production.

In wind energy analysis, novel probabilistic methods were developed to enhance the statistical representation of wind speed, improving both univariate and bivariate analyses [40]. Accurate wind speed modeling is critical for the optimal design and operation of wind-based hybrid systems, particularly when integrated with solar energy sources.

Optimization is not limited to generation systems; it also extends to consumption. A modified COA combined with an Artificial Neural Network (ANN) was used to optimize building energy performance [41]. This approach achieved significant energy savings, showcasing the potential for optimization in both energy generation and consumption aspects of hybrid systems.

In grid-connected systems, the challenge of inter-harmonic distortions in hybrid wind and solar energy systems was addressed through innovative Maximum Power Point Tracking (MPPT) techniques, improving both system efficiency and power quality [42]. These techniques are crucial for ensuring stability in grid-connected renewable systems with fluctuating power outputs.

A broad review of optimization techniques for energy storage and HRES provided insights into a range of metaheuristic algorithms aimed at improving system reliability and efficiency [43]. This work bridges the gap between optimizing energy generation and storage, both essential for the stability of hybrid systems.

Expanding on storage optimization, the Jellyfish Search Algorithm (JSA) was introduced to manage hybrid systems incorporating pumped storage, wind, thermal, and solar photovoltaic (PV) technologies [44]. The JSA demonstrated improvements in system reliability and cost reductions, particularly in large-scale hybrid energy projects.

On the economic side, a techno-economic feasibility study of an autonomous hybrid system for a university building in Saudi Arabia provided insights into the optimal sizing of hybrid systems for institutional applications [45]. This study highlights the economic viability of using renewable energy sources to achieve energy autonomy.

Similarly, the design optimization of off-grid hybrid systems was explored with a focus on building energy performance and climate change [46]. The adaptability of hybrid systems to varying climate conditions is essential for ensuring long-term sustainability, especially in regions with extreme weather conditions.

For remote applications, the Flower Pollination Optimization Algorithm (FPOA) was applied to optimize an off-grid PV-Fuel Cell system [47]. This approach demonstrated the viability of hybrid systems in remote areas with limited access to the main grid, making it a practical solution for rural electrification.

Machine learning techniques also play a role in hybrid system optimization. Statistical methods combined with ANNs were used for decision-making and system design in green energy applications [48]. This integration of optimization and artificial intelligence further enhances system performance and energy efficiency.

In the transportation sector, fuel cell hybrid electric vehicles (FC-HEVs) were optimized through sizing and thermal control strategies [49]. This study illustrated how hybrid renewable technologies can extend beyond stationary energy systems, demonstrating their versatility.

Several studies have focused on rural electrification and agricultural applications. For example, the viability of a PV/Wind/Biomass hybrid system for a small village in Egypt was assessed, providing a case study in rural electrification [50]. Similarly, an economic analysis of renewable energy configurations for remote regions in Saudi Arabia highlighted the importance of hybrid systems in meeting energy needs in isolated areas [51].

In agriculture, hybrid PV-Biomass systems have been proposed to support large-scale irrigation projects in Saudi Arabia [52]. These systems optimize energy inputs, significantly improving resource efficiency and productivity in water-scarce regions.

A hybrid Invasive Weed Optimization and Particle Swarm Optimization (PSO) algorithm was developed to manage biomass/PV micro-grids [53]. This approach improved both system efficiency and reliability, showcasing the potential of advanced optimization techniques in managing complex microgrids.

Existing literature on renewable energy systems shows that the focus has been shifted to hybrid energy sources for reliability and sustainable energy. Several areas are of great importance in this regard. Optimization is the first aspect that focuses on improved convergence and accuracy for such systems and the Cuckoo algorithm, Gaussian model, Vulture optimization, Jellyfish algorithm, flower pollination optimization, etc. have been utilized in the existing research. Another important factor for such systems is to strike a balance between operational costs and emissions for a sustainable environment and AVOA, JSA, ANN, etc. Distortions in HRES are yet another important problem that was focused on by the researchers and MPPT, machine learning models have been prudent solutions. In addition, the PSO algorithm has also been leveraged for biomass micro-grid solutions. Such efforts paved the way for future HRES to obtain efficient, reliable, and sustainable energy systems.

## 3 System description

**Figure 1:**
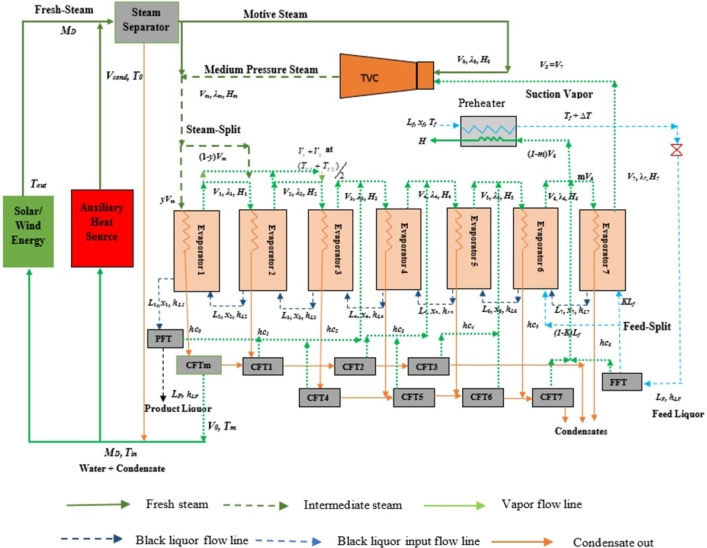
Schematic of proposed hybrid MEE with the integration of Solar/Wind energy field.

The hybrid configuration depicted in this study comprises an LFR solar field, wind generator, and ESSs integrated MEE, as illustrated in Fig. 1. This schematic outlines the integration model aimed at enhancing energy recovery and utilization in the concentration of weak black liquor. The model developed here is an extension of previous work [19], with the sole modification being the incorporation of a wind source to generate the necessary motive steam to operate the entire system. Both solar and wind sources are utilized to operate the unit sustainably, with an auxiliary energy source available to supplement the required energy during renewable energy failures caused by various climatic conditions.

The considered MEE has seven effects in which steam is fed as the heating medium to concentrate the input black liquor, and the outputs are vapor, concentrated liquor, and condensate. The feed liquor enters into the MEE in a backward manner, i.e., to the 7*th* effects of the evaporator train, and the product is obtained from the 1*st* effects. The flow direction of the vapor is gradual from the 1st to the 7*th* effects the output vapor from the 1*st* effects has been treated as the input to the next effects. The incorporated ESSs with the heptad-based MEE are steam-, feed-split, feed preheater, TVC, and flash tanks. The intake steam ($V_{0}$) supplied from the external source passes through the TVC that compresses the vapor out from the 7th effect ($V_{7}$) and produces an intermediate vapor ($V_{m}$) with a temperature of $T_{m}$. Further, this amount of vapor is split into two parts with a split fraction of *y* and (1−y) that enters the 1*st* and 2*nd* effects, respectively. The vapor exits from these effects have been combined with each other and fed to the 3rd effect as the input with an average temperature of ($T_{avg}$).

A preheater has been placed to preheat the feed liquor ($L_{f}$) with a temperature of (*δT*) and a fraction of output vapor from the 6th effects ($V_{6}$) with a vapor fraction of ‘1-*m*’ has been fed as input to this preheater; whereas, *m* fraction of $V_{6}$ has been sent to the 7*th* effects of the MEE. The input liquor has also been split into two parts with a liquor split fraction of *k* and fed to the 7*th* effects and the rest of the liquor has fed to the 6*th* effects along with the liquor out from the 7*th* evaporator ($L_{7}$). The product liquor ($L_{1}$) has been exited from the 1st effect with a temperature the same as the output vapor temperature from that effect. Another output of the evaporator unit is the condensate obtained from each effect of the evaporator with condensate enthalpy *hc*' at a temperature same as that of the input steam/vapor temperature to that effect. The product, feed, and condensates have been fed to the flashing tanks (FTs) to extract the vapor that could be used as additional heat, fed as input for the 4*th* to 7*th* effects in order to make an efficient re-utilization of the waste heat. The arrangement of these FTs (CFT1 to CFT7, PFT, and FFT) has been illustrated in Fig. 1. At Evaporator 1, latent heat is lost due to vaporization, producing some condensate by $V_{m}$. This condensate is flashed by a Condensate Flash Tank (CFT1) to obtain a $V_{0}$ amount of vapor at the same temperature $T_{m}$. The remaining vapor from CFT1 is fed to CFT2 to extract heated vapor for further utilization. Similarly, a series of Condensate Flash Tanks (CFT2 to CFV8) are linked to the feed and product flash tanks (FFT and PFT) to supply extra vapor to the fourth through seventh stages.

As depicted in Fig. 1, renewable fields are utilized to heat fresh water for steam generation, serving as a heating source to operate the evaporation unit. Condensate from the steam separator and Evaporator 1 is also combined with the water source to reduce water intake. The mass flow rate of steam generated by the renewable field is denoted as MD, with temperature variation in the input ($T_{in}$) and output ($T_{out}$). The fresh steam is directed to the steam separator to provide the desired steam flow rate $V_{0}$ to the TVC unit, at temperature $T_{0}$. TVC also utilizes low-pressure steam from Evaporator 7 to generate medium-pressure steam, $V_{m}$ at temperature $T_{m}$, which is used to operate the MEE.

**Figure 2:**
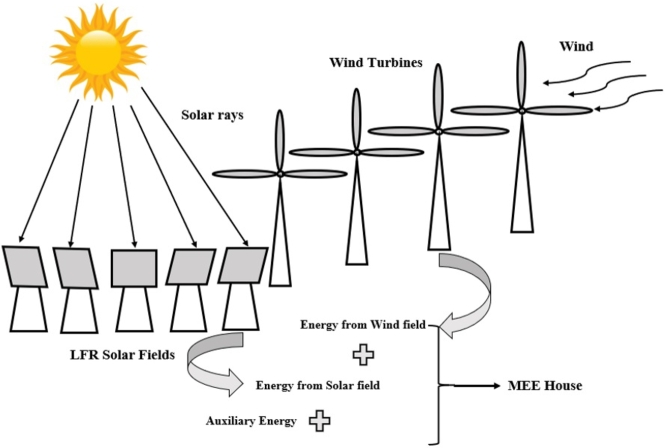
Solar and wind energy integration with the proposed hybrid MEE model.

At Evaporator 1, there is a loss of latent heat due to vaporization, resulting in the production of some condensate by $V_{m}$. This condensate is flashed by a condensate flash tank (CFT1) to obtain a $V_{0}$ amount of vapor at the same temperature $T_{m}$. The remaining vapor from CFT1 is fed to CFT2 to extract heated vapor for further utilization. Similarly, a series of CFTs (CFT2 to CFV8) are linked to the feed and product flash tanks (FFT and PFT) in order to supply extra vapor to the fourth through seventh stages. Additionally, three ESSs (steam- and feed-split, and feed preheating) have been unified by split fractions of *y*, *k*, and *m*, correspondingly, to enhance SE. Fig. 2 illustrates the pre-installation schematic of solar and wind source integration with the proposed hybrid MEE model.

## 4 Approaches and methodologies

The energy models were developed based on appropriate assumptions aligned with the actual system, using fundamental thermodynamic laws. Prime assumptions include

i. All the system parameters are performed under a steady state.
ii. The product concentration of 1st effects from MEE is constant.
iii. The surrounding heat losses are negligible due to well-insulated system components operating at relatively low temperatures.
iv. Overhead vapors are considered pure steam to operate the following effects of MEE.
v. The system operates at ideal operating conditions (without any plant complexities).

With these assumptions, the mass, component, and heat transfer balance for each stage of the proposed MEE model has been generalized and stated as in Equations (1) to (4), respectively.

(1) $$V_{i}=L_{(}i+1)-L_{i}\;\text{for }i=1,2,\ldots ,6$$

(2) $$L_{i}x_{i}=L_{i+1}x_{i+1}\;\text{for }i=1,2,\ldots ,6$$

(3) $$(L_{i}-L_{i-1})(\lambda_{i-1}-hc_{i-1})+L_{i}(H_{i}-h_{i})+L_{i+1}(h_{i+1}-H_{i})=0$$

(4) $$U_{i}A_{i}(T_{i-1}-T_{i})-(L_{i}-L_{i-1})(\lambda_{i-1}-hc_{i-1})=0$$

where, *i* is the count of evaporator units, *V* and *L* represent the vapor and liquor flow rate respectively. However, *hc*, *H*, and *h* represent the latent heat of vaporization, condensate, vapor, and liquor enthalpy respectively. $\lambda_{i}$ is the latent heat of vaporization, *U* and *A* represent the overall heat transfer coefficient and heat transfer area of the evaporator units respectively.

### 4.1 Energy modeling of MEE

The energy models utilized in this study analyze the performance characteristics of ESSs integrated MEEs concerning various associated process parameters. Mass, component, energy balance, and heat balance equations are employed to different effects of both the base (b-) and hybrid (h-) MEEs, resulting in a set of nonlinear steady-state equations [15], [54]. These equations, along with those for condensate flashing, have been thoroughly elucidated in existing literature [20]. Therefore, this study leverages these established models to solve the proposed industrial optimization problem.

### 4.2 Formulation of objective function

This study focuses primarily on maximizing SE while minimizing SC. Given the inverse relationship between SE and SC, maximizing SE inherently leads to reduced SC utilization. Thus, the objective function of the problem can be expressed as the maximization of SE under various optimization constraints, as represented in Equation (5).

(5) $$Max(F(z))=Max(SE)=\frac{\sum_{i=1}^{7}V_{i}}{V_{0}}=\frac{L_{f}-L_{1}}{V_{0}}\;\text{Subjected to }g_{i}(z)=0$$

where F(x) represents the objective function (maximize the SE). SE measures the efficiency of a MEE, representing the amount of water vapor (steam) produced per unit of steam input. Specifically, SE is the ratio of the mass of evaporated water (i.e., $\sum_{i=1}^{7}V_{i}$) to the mass of motive steam ($V_{0}$) supplied to the system. After solving, $\sum_{i=1}^{7}V_{i},L_{f}-L_{1}$ is obtained representing the difference in the amount of feed liquor and product liquor. $g_{i}(z)$ represents the equality constraints obtained from the set of fourteen nonlinear equations of the MEE. The boundaries of the different process parameters such as $V_{0}$, $T_{i}$, (i=1,6), $L_{i}$, (i=1,2,...,7), which will be optimally estimated, taken from the real-time seven effects MEE [6], [14].

The boundaries of the different process parameters such as $V_{0}$, $T_{i}$, (i=1,2,…,6), $L_{i}$, (i=1,2,…,7), which will be optimally estimated, taken from the real-time seven effects MEE [6], [14], which has been represented in Table 1. Finally, the extreme boundaries for Ti and Li are set for smooth conduct of the optimization problem and are represented in Equation (6).

(6) $$T_{i}>T_{\left(i+1\right)},(i=1,3,\ldots ,6)L_{i+1}>L_{i},(i=1,2,\ldots ,6)$$

**Table 1:** Boundary values of unknown process parameters for i${}^{th}$ effects of suggested MEE.

b-MEE
*T*_*i*_(^∘^*C*)=[100:110; 70:85; 66:74 60:70 55:65 52:63]
*L*_*i*_(*kg*/*s*)=[2:5; 3:6; 4.5:7; 6.5:9; 9:11; 10.5:13; 13:15]

**h-MEE**
*T*_*i*_(^∘^*C*)=[100:125; 70:120; 66:90; 60:75; 55:65; 52:63]
*L*_*i*_(*kg*/*s*)=[1.5:5: 3:6; 4:8; 6.5:10.5; 9:12; 6:13; 6:14]

### 4.3 Optimization strategies

This investigation adopts a novel optimization approach called WaOA [36]. WaOA is inspired by various phases of walrus hunting behavior, including feeding, migrating, escaping, and fighting predators. To mathematically model these behaviors, WaOA consists of three main steps: exploration, migration, and exploitation, which aim to provide optimal results. The optimization strategy employed in this investigation aims to maximize SE while minimizing SC, as they exhibit an inverse relationship. The pseudo-code of WaOA is presented in Algorithm 1.

**Algorithm 1:**
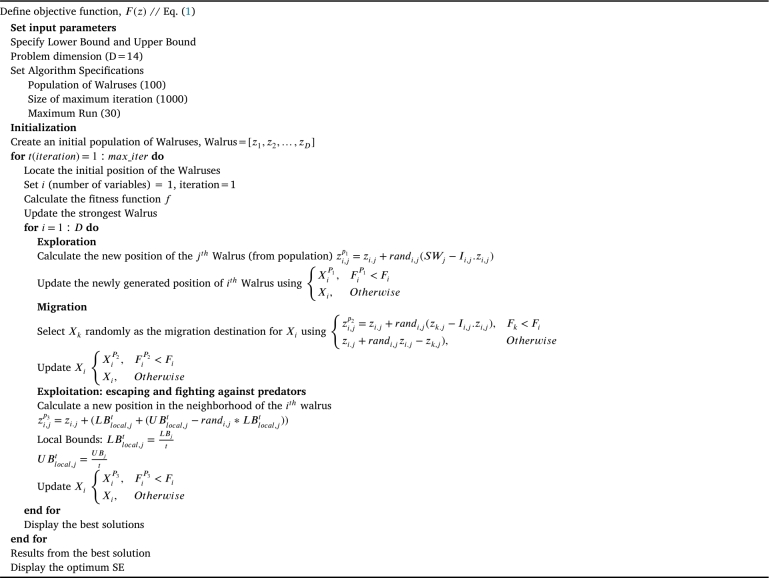
Pseudo code for SE computation using WaOA.

## 5 Renewable energy integration

### 5.1 LFR integration

**Figure 3:**
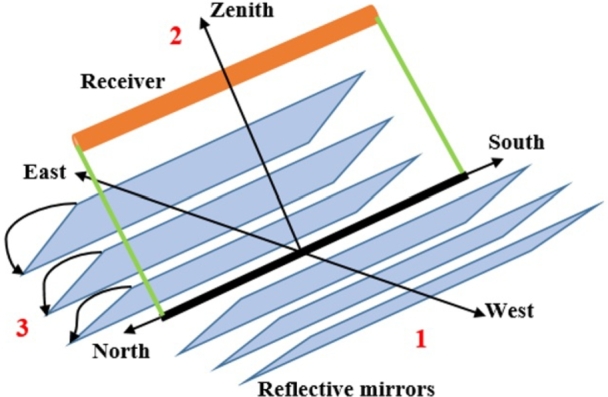
Main components of an LFR: (1) the primary Fresnel Reflectors field, (2) the receiver, and (3) the solar tracking mechanism.

The LFR represents one of the most recent advancements in CSP technology, gaining popularity in recent decades [55]. An LFR-based power plant consists of a series of long, narrow mirrors with shallow curvature and linear receivers positioned above them. These mirrors employ a simple line-focus geometry with single-axis tracking to concentrate sunlight. Additionally, a small parabolic mirror is affixed on top of the receiver to further concentrate sunlight, as illustrated in Fig. 3 [56].

### 5.2 Energy modeling of LFR

The energy model of the LFR is designed to calculate both the amount of energy generated and the outlet temperature of the collector in series [9], [57]. The integration of the LFR field is a supplement to conventional energy source utilization for the MEE operation. The expression for the per unit heat gain of the LFR solar collector is given by Equation (7).

(7) $$q=\eta .DNI.IAM-B_{l}\left(\frac{T_{out}+T_{in}}{2}-T_{a}\right)=\eta .DNI.IAM-U_{l}\Delta T_{avg}=\eta I_{D}-U_{l}\Delta T_{avg}$$

where $\Delta T_{avg}$ and $I_{D}$ represent the solar collector and atmospheric temperature difference and the effective design radiation respectively. The LFR aperture area can be determined by the Equation (8).

(8) $$A_{LFR}=\frac{Q_{MEE}}{q_{D}}=\frac{Q_{MEE}}{\eta I_{D}-U_{l}\Delta T_{avg}}$$

The LFR solar collector mitigates the heat demand of the MEE at the desired DNI ($I_{D}$). Whereas, at reduced DNI (*I*), auxiliary heating is essential to generate a limited quantity of steam. The quantity of auxiliary heat needed at reduced DNI is expressed by the relation Equation (9) [58].

(9) $$Q_{aux}(I)=Q_{MSE}-q(I)A_{LFR}$$

Putting the relation in Equation (9), the relation of auxiliary heat required can be expressed as Equation (10).

(10) $$Q_{aux}(I)=\eta (I_{D}-I)A_{LFR},\forall I_{D}>I$$

The input operational parameters of the LFR solar field have been obtained from data obtainable from Jodhpur, India [19] and depicted in Table 2.

**Table 2:** Design parameters of LFR*.

Parameter(s)	Values(s)
Optical efficiency of LFR (*η*)	0.65
Heat loss Coefficient of LFR (*Ul*)	0.65 W/m^2^ K
Specific land required	2 *A*_*p*_
IMA effect	Novatech design [59]
Collector outlet dryness (*DF*)	0.5
Cut off Radiation (*I*_*c*_)	100 W/m^2^
Maximum Design Radiation (*I*_*Dmax*_)	600 W/m^2^
Plant capacity (*P*_*D*_)	1 MWe
Aperture area of LFR	17,410 m^2^
Lifetime	25 years

* Data available from Jodhpur, India.

### 5.3 Weibull modeling of LFR

Solar irradiance is a random stochastic source of energy and solar power takes a nonlinear relation through it. Weibull distribution is implemented for processing the solar irradiance time series data. The probability distribution function (pdf) of solar irradiance is expressed in Equation (11)

(11) $$pdf(i)=\frac{\alpha }{\beta }{\left(\frac{i}{\beta }\right)}^{\alpha -1}exp\left[-{\left(\frac{i}{\beta }\right)}^{\alpha }\right]$$

Finally, all the solar power generating unit's output has to be taken in the manner of one random variable and the cumulative distribution function (*cdf*) for forecasted solar irradiance will be calculated as per the equation shown in Equation (12).

(12) $$cdf(i)=1-e^{\left[-{\left(\frac{i}{\beta }\right)}^{\alpha }\right]}$$

where, i≥0 is the randomly measured solar irradiance ($kW/m^{2}$) and α,β≥0 are representing the shape and scale parameters of solar irradiance data respectively. The solar power ($P_{solar}$) will be computed from forecasted solar irradiance time series data. Solar power is represented in Equation (13) [60].(13)
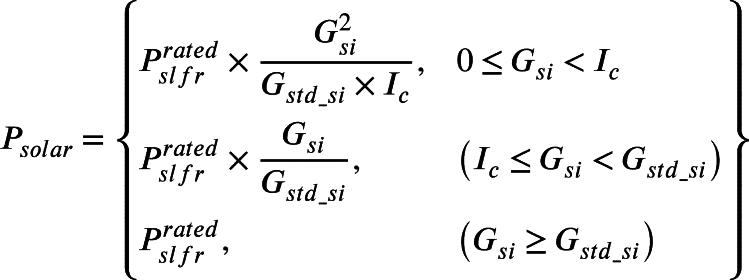
 where $P_{solar}$ represents the power generated by solar LFR, $G_{si}$ and $G_{std\_si}$ represents the global and standard solar irradiance respectively. $P_{slfr}^{rated}$ and $I_{c}$ represents the rated capacity of solar LFR and certain solar irradiance points respectively.

For solar irradiance, the shape parameter (*α*) indicates the variability of solar energy; a higher *α* value signifies more stable solar irradiance, whereas a lower *α* suggests greater fluctuations. The scale parameter (*β*) represents the average level of solar irradiance, with higher values indicating greater potential for energy generation. This information is vital for designing solar technologies like LFR to maximize efficiency.

Similarly, for wind speed, the shape parameter (*α*) reveals the distribution's skewness, affecting the optimal placement of wind turbines. A higher *α* suggests more consistent wind speeds, while a lower *α* indicates variability. The scale parameter (*β*) represents the average wind speed, with higher values corresponding to increased energy production potential. Accurate estimation of these parameters is essential for effective wind turbine selection and placement. By integrating Weibull parameters into the design and optimization of solar and wind energy systems, the study ensures that systems are tailored to local conditions, enhancing reliability and efficiency. This comprehensive approach minimizes waste and maximizes energy production, contributing to more stable and efficient energy systems. The revised manuscript incorporates these explanations to better connect the findings with their impact on energy system performance.

### 5.4 Techniques for estimation of solar irradiance data and power

#### 5.4.1 Solar irradiance data collection

**Figure 4:**
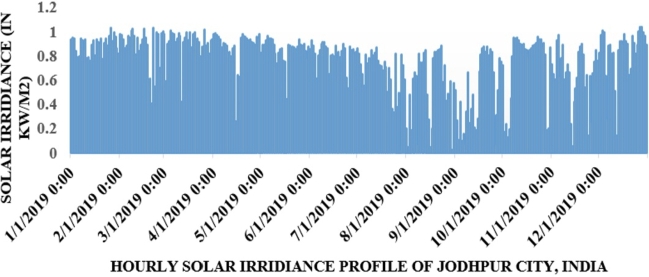
Hourly solar irradiance (kW/m^2^) of Jodhpur City, from January to December 2019.

**Figure 5:**
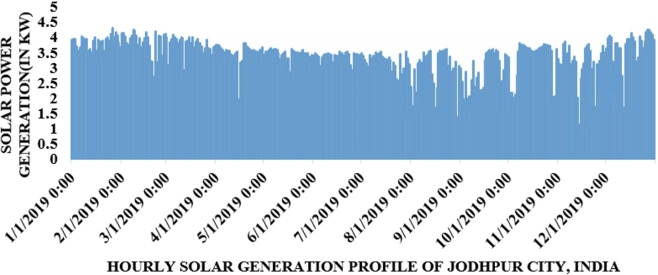
Hourly wind power generation (kW) of Jodhpur, India from January to December 2019.

Jodhpur City solar irradiance data has been taken from the National Institute of Solar Energy (NISE), India in time series format. In Fig. 4 hourly solar irradiance profile of Jodhpur City, India is depicted from January to December 2019. Hourly solar power generation profile is shown in Fig. 5.

#### 5.4.2 Techniques for estimation of solar power

Several approaches have been implemented for determining the two constraints of Weibull distribution. One of these techniques is proposed by Steven and Smulders known as the modified likelihood technique (MLT) [61]. The above data is processed in frequency distribution format for calculating the Weibull parameters of solar irradiance in MLT [62]. MLT requires larger nonlinear complex mathematical iterations. In this technique, *α* and *β* represent the shape parameter and scale parameter of solar irradiance, respectively. These parameters control the form and spread of the probability distribution, and they are used to describe the statistical behavior of solar irradiance over a given period, which is determined by Equation (14) and (15), respectively.

(14) $$\alpha ={\left[\frac{\sum_{sr=1}^{NR}i_{sr}^{\alpha }ln(i_{sr})}{\sum_{sr=1}^{NR}i_{sr}^{\alpha }}-\frac{\sum_{sr=1}^{NR}ln(i_{sr})}{Nr}\right]}^{-1}$$

(15) $$\beta ={\left[\frac{1}{NR}\sum_{sr=1}^{NR}i_{sr}^{\alpha }\right]}^{1/\alpha }$$

where $i_{sr}$ is the solar irradiance in each sunny hour. *NR* and *sr* are the number of nonzero points available from solar irradiance data. For

• α>1, the distribution has a peaked shape, indicating that the solar irradiance values are more concentrated.
• α=1, the Weibull distribution becomes an Exponential distribution, which suggests that solar irradiance decays exponentially over time.
• α<1, the distribution has a more spread-out shape, implying that solar irradiance values vary widely with less concentration around a mean value.

Whereas, *β* represents the characteristic scale of the irradiance values, often related to the average intensity of solar radiation over time, i.e., a larger *β* corresponds to higher values of solar irradiance, while a smaller *β* vice versa. By adjusting the *α* and *β* parameters, the distribution can accurately reflect the statistical characteristics of solar energy at different sites or times of year.

**Figure 6:**
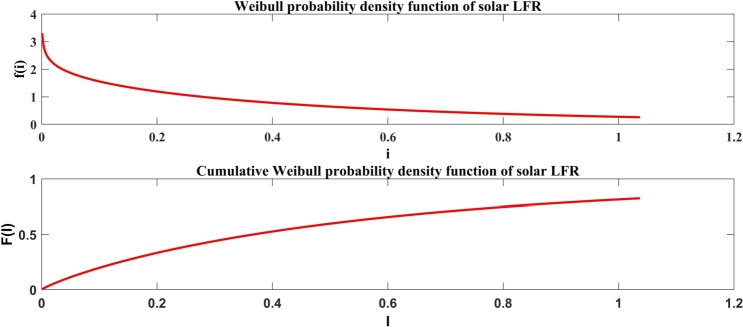
Solar irradiance for sample periods, (Top) Weibull pdf, and (Bottom) Cumulative Weibull pdf.

Fig. 6 specifies the Weibull *pdf* of solar irradiance and the cumulative Weibull *pdf* of solar irradiance of Jodhpur city for January to December 2019.

Modified MLT (MMLT) is implemented when solar irradiance data is available in frequency distribution arrangement. In the case of this MMLT, the Weibull parameters of solar irradiance data are calculated using Equations (16) and (17).

(16) $$\alpha ={\left[\frac{\sum_{sr=1}^{NR}i_{sr}^{\alpha }ln(i_{sr})p(i_{sr})}{\sum_{sr=1}^{NR}i_{sr}^{\alpha }P(i_{sr})}-\frac{\sum_{sr=1}^{NR}ln(i_{sr})p(i_{sr})}{P(i_{sr}\geq 0)}\right]}^{-1}$$

(17) $$\beta ={\left[\frac{1}{p(i_{sr}\geq 0)}\sum_{sr=1}^{NR}i_{sr}^{\alpha }p(i_{sr})\right]}^{1/\alpha }$$

$p(i_{sr})$ represents the frequency of the solar irradiance within bins and $P(i_{sr}\geq 0)$ represents the probability of occurring the solar irradiance greater than equal to zero i.e., nonzero solar irradiance.

**Figure 7:**
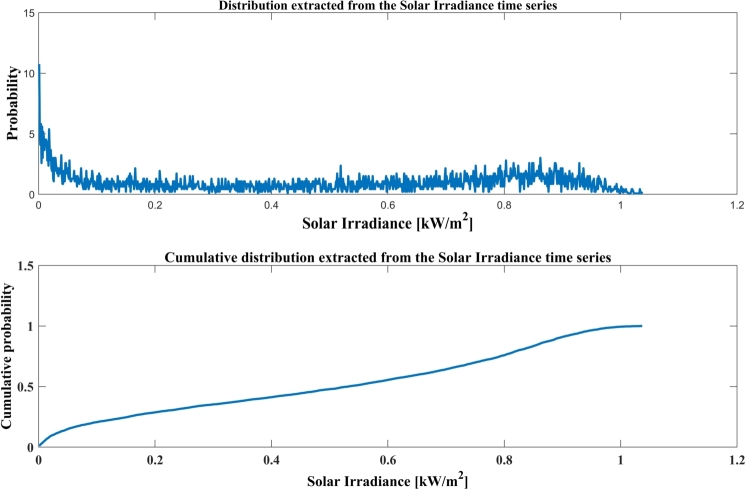
Distribution and cumulative distribution extracted from solar irradiance data.

Fig. 7 illustrates the distribution extracted from solar irradiance time series data and Cumulative distribution extracted from solar irradiance of Jodhpur City from January to December 2019 time periods. Table 3 illustrates the Weibull Parameters of solar irradiance for mathematical models: A case study of Jodhpur, India.

**Table 3:** Weibull parameters of solar irradiance for mathematical models.

Mathematical Models		MLT	MMLT
Weibull Parameters of solar irradiance data	*α*	1.88	1.74
	*β*	3.55	3.42

## 6 Wind energy

### 6.1 Modeling of wind source

Wind turbines transform the mechanical energy of wind into electrical energy. This process involves the kinetic energy of the wind being transmitted through a system of aerodynamic blades to drive an electrical generator, producing electric energy. Wind turbines are primarily classified into two categories based on the orientation of the spin axis: vertical and horizontal axis turbines. Horizontal-axis wind turbines are the most commonly used for electricity generation and are therefore considered in this investigation for the overall system design. Unlike the LFR (used for direct steam generation), the wind turbines use the generated electricity to heat up the water and produce the steam that will lead to operating the MEE.

### 6.2 Mathematical modeling for wind energy calculations

The velocity of wind is a random stochastic variable in nature and wind power conveys a nonlinear relation through it. The wind speed measured from Jodhpur city, India is considered here for this modeling and simulation. Weibull distribution is implemented for processing the wind power and wind speed. The pdf of wind speed is expressed in Equation (18)

(18) $$pdf(v)=\frac{u}{\alpha }{\left(\frac{v}{\alpha }\right)}^{\mu -1}exp\left[-{\left(\frac{v}{\alpha }\right)}^{\mu }\right]$$

The wind power $P_{wi}$ will be computed from forecasted wind speed and this is also stochastic in nature. This wind power is represented in Equation (19).(19)
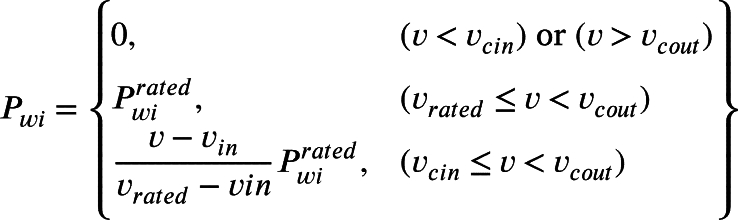


When the wind speed is in the mid of the $v_{rated}$ and $v_{cin}$ then wind farm power generation will be assumed constant. Finally, all the wind power generating unit's output has to be taken in the manner of one random variable $P_{wi}$, and pdf for this will be as in Equation (20).

(20) $$pdf(p_{wi})=\frac{\mu \gamma \times v_{in}}{\alpha \times p_{wi}^{rated}}{\left[\frac{\left(1+\frac{\gamma p_{wi}}{p_{wi}^{rated}}\right)v_{in}}{\alpha }\right]}^{\mu -1}\times exp{\left[-\frac{\left(1+\frac{\gamma p_{wi}}{p_{wi}^{rated}}\right)v_{in}}{\alpha }\right]}^{\mu }$$

where $\gamma =\left(\left(\frac{v_{rated}}{v_{cin}}\right)-1\right)$.

### 6.3 Techniques for estimation of wind power and wind speed data

#### 6.3.1 Wind speed data collection

**Figure 8:**
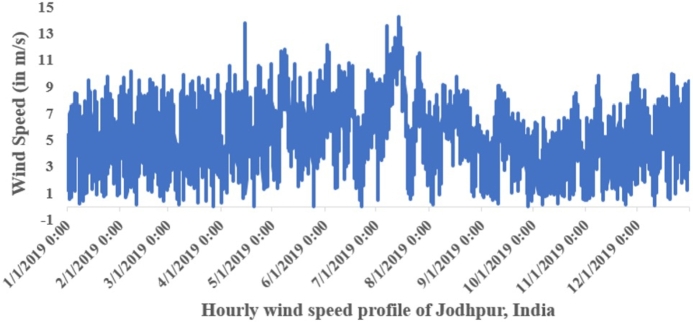
Hourly wind speed (m/s) from January to December 2019.

**Figure 9:**
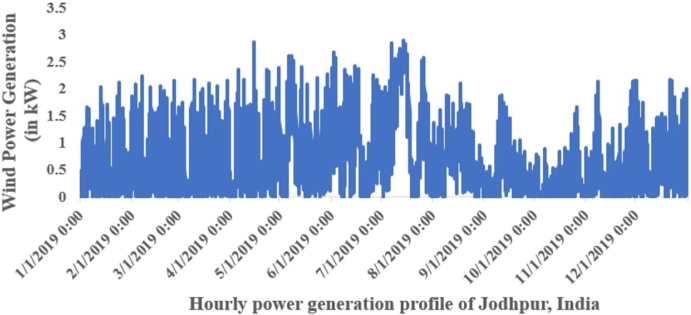
Hourly wind power generation (kW) from January to December 2019.

Wind speed data for Jodhpur city, provided by the National Institute of Wind Energy (NIWE), India, has been transformed into a frequency distribution format to calculate Weibull parameters using MLT. Fig. 8 displays the hourly wind speed profile from January to December 2019 for Jodhpur, India, while Fig. 9 illustrates the corresponding hourly wind power generation profile.

#### 6.3.2 Techniques for estimation of wind power

**Figure 10:**
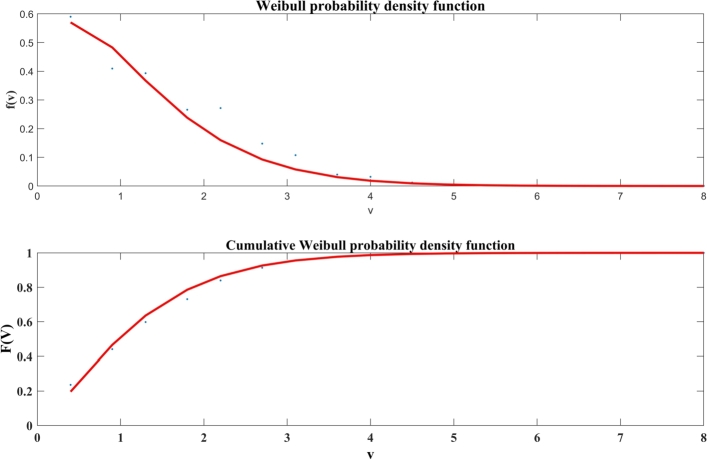
Weibull pdf and Cumulative Weibull pdf for Jodhpur wind profile sample periods.

Various techniques, including MLT proposed by Steven and Smulders [61], have been suggested for determining the two constraints of the Weibull distribution of wind speed. MLT requires large-scale nonlinear mathematical iterations, making it computationally complex. In this technique shape parameter (*φ*) and scale parameter (*c*) of wind speed data are determined by Equations (21) and (22), respectively.

(21) $$\varphi ={\left[\frac{\sum_{s=1}^{N}v_{s}^{\varphi }ln(v_{s})}{\sum_{s=1}^{N}v_{s}^{\varphi }}-\frac{\sum_{s=1}^{N}ln(v_{s})}{N}\right]}^{-1}$$

(22) $$c={\left[\frac{1}{N}\sum_{s=1}^{N}v_{s}^{\varphi }\right]}^{1/\varphi }$$

where, $v_{s}$ represents the wind speed during each hour, *s* and *N* are the numbers of nonzero points of wind data. Fig. 10 indicates the Weibull *pdf* of wind speed and the Cumulative Weibull pdf of wind speed for the Jodhpur city wind profile given for the January to December 2019 time periods.

**Figure 11:**
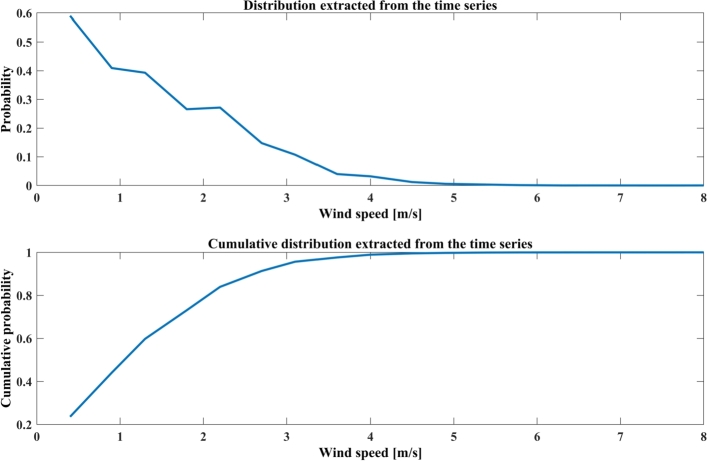
Distribution extracted from wind data and Cumulative distribution extracted from wind data for Jodhpur wind profile.

MMLT is adapted when wind speed data are available in frequency distribution format. In this method, the Weibull parameters of wind speed are calculated using Equations (23) and (24).

(23) $$\varphi ={\left[\frac{\sum_{s=1}^{N}v_{s}^{\varphi }ln(v_{s})P(v_{s})}{\sum_{s=1}^{N}v_{s}^{\varphi }P(v_{s})}-\frac{\sum_{s=1}^{N}ln(v_{s})P(v_{s})}{P(v_{s}\geq 0}\right]}^{-1}$$

(24) $$c={\left[\frac{1}{P(v_{s}\geq 0)}\sum_{s=1}^{N}v_{s}^{\varphi }P(v_{s})\right]}^{1/\varphi }$$

where $v_{s}$ represents the speed of the wind central bins for a specific hour *s* and *N* is the number of bins, $P(v_{s})$ represents the frequency of the wind speed within bins, and $P(v_{s}\geq 0)$ represents the probability of occurring the wind speed greater than equal to zero i.e., nonzero wind speed. In Fig. 11, the distribution extracted from wind speed data and the cumulative distribution extracted from wind speed data for the Jodhpur city wind profile has been given for January to December 2019.

Graphical technique is obtained from the cumulative distribution function. In this approach, the wind speed data is interpolated using a straight line. This method adapts the concept of least square regression [63]. The probability of wind speed occurrence has been expressed in Equation (25).

(25) $$P(v<v_{s})=P(v\geq 0)\{1-exp{\left[-\frac{v_{s}}{c}\right]}^{\varphi }\}$$

Here $P(v<v_{s})$ represents the probability of occurrence of wind speed less than $v_{s}$ and P(v≥0) represents the probability of occurrence of wind speed greater than and equals zero. Equations (25) can be modified as Equation (26).

(26) $$ln\{-ln[1-P(v<v_{s})]\}=\varphi lnv_{s}-\varphi lnc$$

Here a graph between $ln\{-ln[1-P(v<v_{s})]\}$ versus $ln(v_{s})$ is plotted for calculating *k* and *c*. This plot gives a straight line having slope as *k* and *y* intercept as −φlnc.

**Figure 12:**
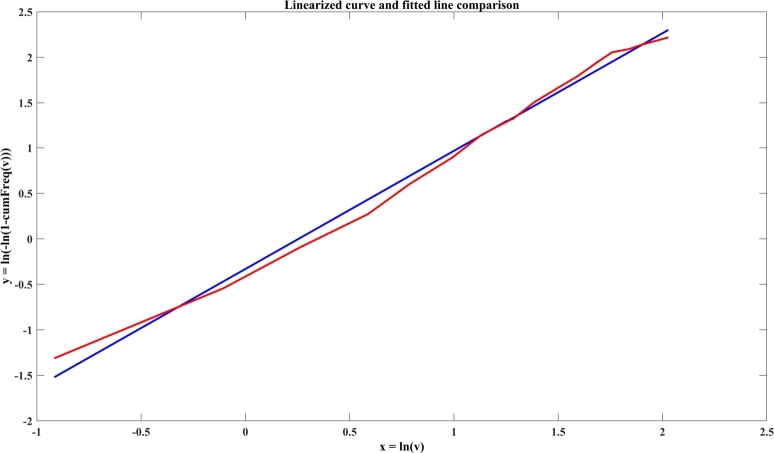
Linearized curve and fitted line comparison of Jodhpur wind speed data.

In Fig. 12, a linearized curve and fitted line comparison of Jodhpur wind speed data is depicted. Table 4 indicates the design parameters of the wind turbine and Table 5 shows the Weibull Parameters of the wind source to calculate the amount of power that the wind turbines generate, it is noticed that the MMP method provides the most suitable parameters and hence is considered for further study.

**Table 4:** Design parameters for wind source (VESTAS V90 2.0 MW Wind Turbine).

Parameter (s)	Values (s)
Rated power	2000 kW
Rotor diameter	90 m
Length of the blade	44 m
Maximum chord of the blade	3.9 m
Swept area of rotor	6362 m^2^
Generator efficiency	90%
Converter efficiency	80%
Inverter efficiency	80%
Frequency	50 Hz
Cut-in speed	4 m/s
Cut-out speed	25 m/s
Average speed	14.5 m/s
Blade diameter	70.34 m
Height	80 m
Lifetime	20 years
Latitude	26.1279^∘^N
Longitude	73.1144^∘^E

Data available from Jodhpur, India.

**Table 5:** The Weibull parameters for the average wind speed.

Mathematical models		MLT	MMLT	Graphical technique
Weibull Parameters	*φ*	1.30	1.24	1.35
	*c*	3.87	3.80	3.92

### 6.4 Wind energy calculations

The wind energy is converted to mechanical energy with a performance coefficient $c_{PR}$. This mechanical energy is conveyed to the generator through a machine-driven transmission with efficiency $\eta_{m}$ (fraction of the power transmitted by the turbine blades to the generator) and generator efficiency $\eta_{g}$ [18]. The overall efficiency of the performance parameters is given in Equation (27).

(27) $$c_{P}=c_{PR}\times \eta_{m}\times \eta_{g}$$

Expected values of performance coefficients are $c_{PR}$=0.45, $\eta_{m}$=0.95 and $\eta_{g}$=0.90 for getting an overall efficiency of 38.475%. The real value of efficiency possibly lies between 25% and 30% and this for due to assumptions of no loss [18]. The kinetic power of the wind turbine is termed the total power of the wind. Hence, the total available wind power is expressed as in Equation (28).

(28) $$P_{m}=c_{P}\frac{\rho }{2}A_{r}v_{s}^{3}$$

where $\rho_{a}$ represents the air density, $A_{r}$ is the rotor area of and $v_{s}$ is the average wind speed. According to the Weibull distribution function, the average output power of a wind turbine is given by Equation (29).

(29) $$P_{avg}=\int_{0}^{\infty }P_{m}P(v_{s})dv_{s}$$

where $P_{m}$ is the maximum output power from the wind turbine at average wind speed $v_{s}$ and $P(v_{s})$ denotes the Weibull distribution function. Considering negligible generator and gearbox losses of the wind turbines and control system are in the form of heat, the quantity of net heat generated from the wind source per unit time is the same as that of the average power ($P_{avg}$).

### 6.5 Renewable integration with system components

The energy analysis with the integration of renewable sources is performed by employing the first law of thermodynamics to the system input. The energy balance equations due to this inclusion for the system components are represented as Equations (30) to (32).

(30) $$Q_{MSE}=M_{D}(h_{out}-h_{in})$$

(31) $$M_{D}h_{out}=V_{0}H_{0}+V_{cond}hc_{ss}$$

where $hc_{ss}$ is the steam separator condensate enthalpy.

Equations (30) and (31) represent the MEE unit and steam separator, respectively while Equation (32) is for the LFR/wind.

(32) $$M_{D}=V_{0}\frac{H_{0}-hc_{ss}}{h_{out}-hc_{ss}}$$

The expressions for the $T_{out}$ and $T_{in}$ for the renewable fields are given by Equations (33) and (34), respectively.

(33) $$T_{out}=T_{0}+\Delta T_{d}$$

(34) $$T_{in}=\frac{(M_{D}-V_{0})T_{0}-V_{0}T_{m}}{M_{D}}$$

Hence, these parameters remain unchanged for both LFR and Wind energy sources.

## 7 Results

**Figure 13:**
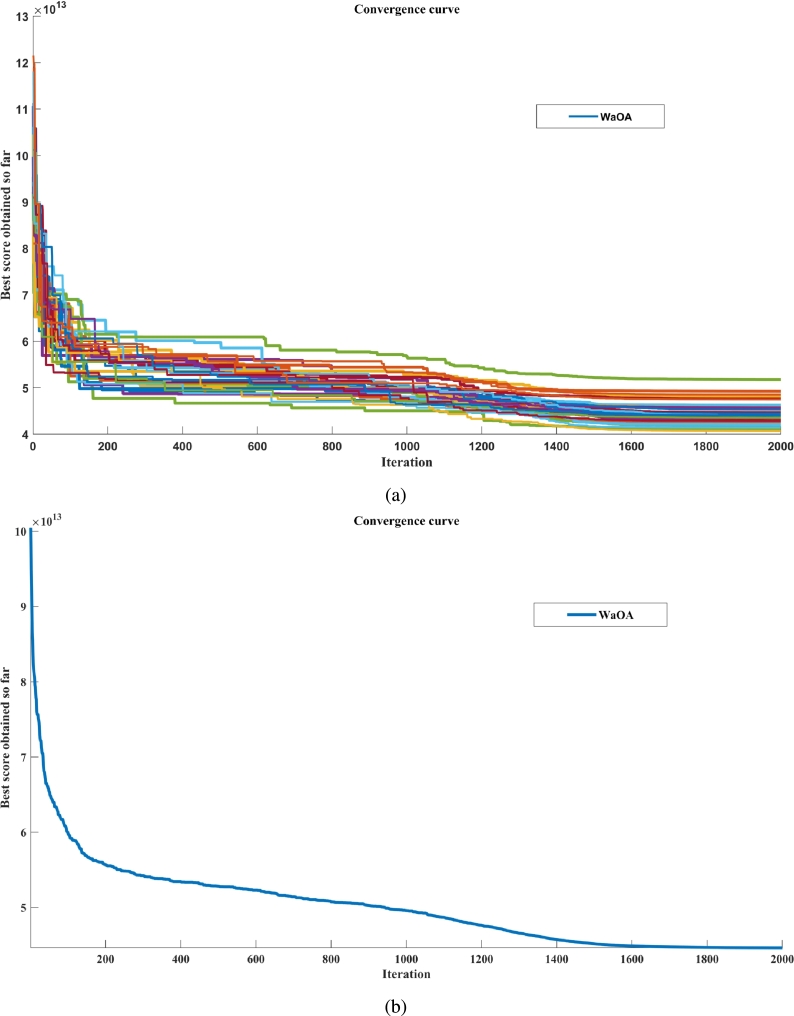
Convergence curve of WaOA for, **(a)** 30 runs, and **(b)** Best run.

The system of equations for the backward feed MEE operating in an Indian pulp and paper industry has been simulated and validated using a state-of-the-art optimization algorithm, WaOA, within the MATLAB environment. The convergence curve of WaOA for this industrial problem is illustrated in Fig. 13, demonstrating the efficiency of the proposed algorithm. The proposed algorithm has been applied to the base MEE model with 14 decision variables, utilizing 2000 maximum iterations and a population of 100, executed 30 times. Statistical analysis of the algorithm yielded an optimal SE value of 5.0297 at the 26${}^{th}$ execution, with an SC of 2.24 kg/s, and an execution time of 73.161 seconds. This performance highlights the algorithm's effectiveness in optimizing MEE systems, demonstrating both high efficiency and reliability. Comparing these results with the previously reported algorithms the execution speed is better compared to Teaching Learning Based Optimization (TLBO) and Artificial Electric Field Algorithm (AEFA) whereas, it produces better energy optimization results in terms of SE and SC than PSO, DEA, TLBO, GWO, and AEFA as reported in [35].

Table 6 displays the optimized process parameters and enthalpy values for both the base (b-) and hybrid (h-) MEE models obtained through simulation, allowing for comparative analysis. It is observed from Table 7, that the vapor, liquor, and condensate enthalpies have been decreased with a decrease in temperature, whereas, the latent heat of vapor increases with increasing in temperature.

**Table 6:** Obtained results of b- and h-MEE using WaOA.

Variables	Unit	Evaporator Effect Numbers							
		1	2	3	4	5	6	7	Output
b-MEE Model									
*T*	^∘^C	147	110	71.647	68.852	62.996	58.983	55.383	52
*λ*	kJ/kg	2109.5	2222.7	2329.0	2336.3	2351.4	2361.5	2370.6	2379.0
*H*	kJ/kg	2749.1	2689.0	2626.1	2621.8	2611.8	2605.2	2599.3	2593.7
*hc*	kJ/kg	637.28	470.76	302.1	289.96	264.6	247.28	231.78	NA
*h*	kJ/kg	329.1	237.14	229.99	228.65	220.9	211.54	201.33	NA
*L*	kg/s	4.42	5.586	6.457	8.451	10.561	12.359	13.892	NA
*V*	kg/s	2.24	1.166	0.871	1.994	2.11	1.798	1.533	1.719
*SC*	kg/s	**2.2400**							
*SE*		**5.0300**							

h-MEE Model									
*T*	^∘^C	120	110.002	80.457	71.81	65.23	61.64	56.34	52
*λ*	kJ/kg	2193.2	2222.7	2305.6	2328.7	2345.6	2354.8	2368.2	2379.0
*H*	kJ/kg	2705.3	2689.0	2640.6	2626.4	2615.5	2609.6	2600.8	2593.7
*hc*	kJ/kg	515.4	470.77	340.49	302.8	274.27	258.75	235.9	NA
*h*	kJ/kg	329.11	258.27	247.33	236.76	230.85	215.2	201.33	NA
*L*	kg/s	4.4947	5.9991	7.341	9.565	11.441	12.989	12.734	NA
*V*	kg/s	2.6277	1.504	1.3419	2.224	1.876	1.548	1.306	1.316
*SC*	kg/s	**1.3160**							
*SE*		**8.4468**

**Table 7:** Vapor extracted from different FTs and feed sections.

Model	Flash ranks	CFTR	PFT	CFT1	CFT2	CFT3	CFT4	CFT5	CFT6	CFT7	FFT
b-MEE	Amount of vapor exit (kg/h)	571.13	368.6	304.81	32.95	145.01	104.69	277.92	384.02	420.88	323.18
h-MEE		203.63	350.11	140.18	42.43	187.92	155.48	319.12	408.78	434.45	323.18

Fed to effects		LFR	4th	4th	5th	6th	4th	5th	6th	7th	7th

**Figure 14:**
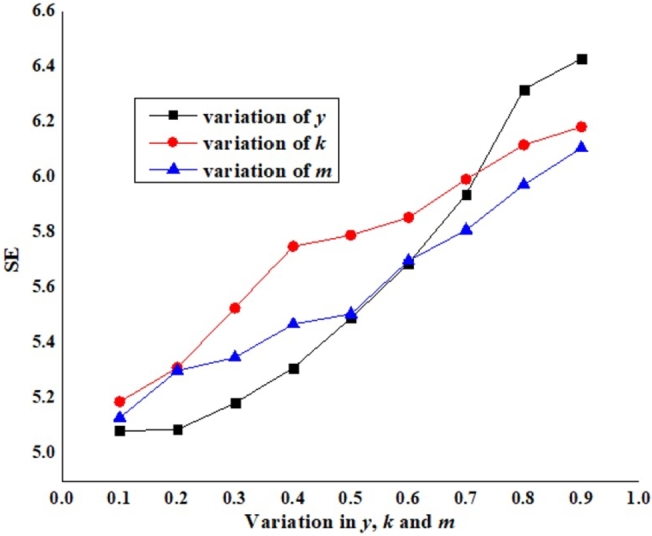
Effect of split fraction variation on SE.

Fig. 14 illustrates the impact of varying split fractions on SE, showing that higher split fraction values (ranging from 0.1 to 0.9) contribute to a better steam economy. This highlights the potential for optimizing MEE performance by adjusting the split fraction to achieve a better steam economy. In this simulation, EM-1 represents the base model without any ERSs with a SE of 4.849 and SC of 2.7 (kg/s). EM-16 (a hybrid of all the ERSs) shows the best result of SE (8.5473) with a SC of 1.6509 (kg/s). However, comparing the results of EM-1 and EM-16, there is an enhancement of 76.27% in SE and a 38.85% reduction in SC.

The proposed models incorporate waste heat reutilization, thereby enhancing SE while maintaining a constant SC through the use of FTs. The quantity of vapor collected by utilizing the waste heat from various flash tanks and corresponding feeding effects are depicted in Table 7. The amount of vapor produced is added according to the feed effect. The simulated result indicates that there is an approximated 2429.73 kg/h and 2361.65 kg/h waste steam extracted from the flash tanks for the b- and h-MEE respectively at a constant SC as observed from Table 7.

**Figure 15:**
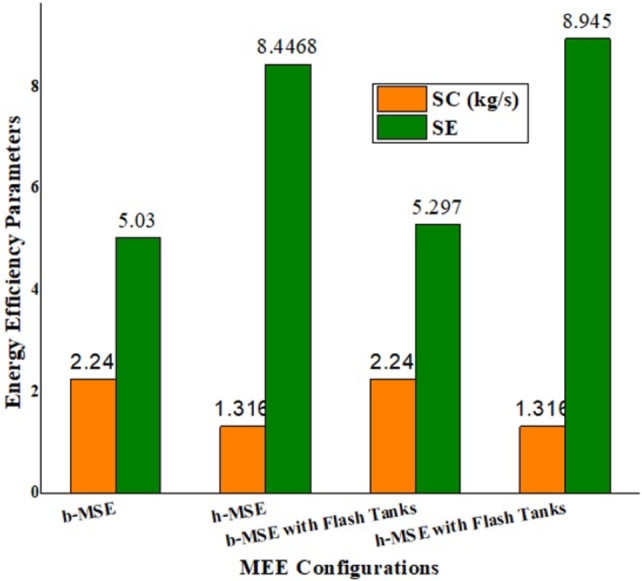
Energy efficiency parameter comparison for proposed MEEs without and with FTs.

Additionally, Table 8 confirms SE calculations for the proposed MEE models: for the base MEE, SE is improved by 5.31%, and for the hybrid MEE, it is enhanced by 5.89% with a one-time model modification. Fig. 15 presents a comparative analysis of energy efficiency parameters for the proposed MEE models without and with FTs. The first two columns represent SC and SE without FTs, while the last column indicates the improved results achieved with FTs. The first two columns represent SC and SE without FTs, while the last column indicates the improved results achieved with FTs, i.e., an overall 73% SE enhancement obtained without extra consumption.

**Table 8:** Additive vapors and SE calculation due to incorporation of FTs for the proposed MEE.

Model	Parameters	Extracted vapor added to respective effects						
		1${}^{st}$	2${}^{nd}$	3${}^{rd}$	4${}^{th}$	5${}^{th}$	6${}^{th}$	7${}^{th}$
b-MEE	Vapor added (kg/h)	0	0	778.1	310.87	596.7	744.06	0
	Exit vapor (kg/h)	4197.6	3135.6	7178.4	7596	6472.8	5518.8	6188.4
	Total vapor(kg/h)	4197.6	4140	7956.5	7906.87	7069.5	6262.86	6188.4
	SE	**5.297**						

h-MEE	Vapor added (kg/h)	0	0	645.77	361.55	596.7	757.63	0
	Exit vapor (kg/h)	5414.4	4830.8	8006.4	6753.6	5572.8	4701.6	4737.6
	Total vapor(kg/h)	5414.4	4830.8	8652.17	7115.15	6169.5	5459.23	4737.6
	SE	**8.945**

### 7.1 Synthesis of renewables integration

The proposed MEE models are based on steam analysis, providing insight into the thermal behavior of various streams within the system. Steam for evaporation is sourced from auxiliary heating units using conventional energy. Incorporating an LFR solar field-based CSP plant can reduce reliance on conventional energy. Table 9 presents calculated parameters for the MEEs with the LFR solar field. The heat obtained from the LFR field for the base (b-) and hybrid (h-) MEE models is 4730.7 kW and 2919.4 kW, respectively. Two reduced apertures effective DNI cases are considered to assess auxiliary heat requirements in the presence of the LFR as demonstrated in Table 9. Results indicate as demonstrated in Table 9 that wind integration performs better in meeting heat demands at average wind speeds, while deviations are observed during cut-in and cut-out wind speeds. Auxiliary heat sources are required for both b- and h-MEEs at cut-in wind speeds, whereas wind energy generation exceeds demand at average and cut-out wind speeds, offering surplus energy for utilization.

**Table 9:** Estimated renewables and auxiliary parameters for the proposed MEEs.

Model	Parameters	*M*_*k*_ (kg/s)	*T*_*in*_ (^∘^C)	*T*_*out*_ (^∘^C)	Solar energy			Auxiliary energy			
					*Q*_*MEE*_ (kW)	*A*_*LFR*_ (m^2^)	*q*_*d*_ (kW)	*q* (kW)		$Q_{Aux}$ (kW)	
								(*I*=500 W/m^2^)	(*I*_*c*_=194 W/m^2^)	(*I*=500 W/m^2^)	(*I*_*c*_=194 W/m^2^)
b-MEE	Values acquired	3.286	147	157	4730.7	17.62	268.45	248.95	50.05	343.62	3848.5
h-MEE		1.917	133.5	157	2919.4	10.7	272.84	253.34	54.44	208.66	2336.9

Wind energy											

*v* (m/s)					$P_{avg}$ (kW)			$Q_{MEE}$ (kW)		$A_{Aux}$ (kW)	

b-MEE	Values acquired	4 (Cut-in)			1017.705			4730.7		3712.995	
		14.5 (Average)			76385.06					-	
		25 (Cut-out)			394899.77					-	
h-MEE		4 (Cut-in)			1017.705			2919.4		1901.695	
		14.5 (Average)			76385.06					-	
		25 (Cut-out)			394899.77					-	

### 7.2 Comparative analysis with existing works

The efficacy of our proposal hinges on a thorough comparative analysis of diverse MEE models documented in prior literature. This comparative scrutiny specifically centers on MEE configurations employing seven effects. Table 10 states the comparative analysis.

**Table 10:** Comparative analysis of proposed models with previous literature.

Ref.	Flow sequence	Interested ESSs	Solving technique	Best energy efficiency values	
				SC (kg/h)	SE
[15]	Backward	Steam split, FTs, vapor bleeding	Generalized cascade algorithm	8776	5
	Mixed			8881.2	4.94

[64]	Backward	Steam split, FTs, vapor bleeding	Modified temperature path	7895	5.56

[29]	Backward	Steam-, Feed-split, Feed preheater	Interior Point Method	7092	6.49

[30]	Backward	Steam-, Feed-split, Feed preheater, FTs	Genetic Algorithm, Differential Evolution, Particle Swarm Optimization	5945.4	8.65

[31]	Backward	Steam-, Feed-split, Feed preheater	Water Cycle Algorithm	6908.4	7.09

[20]	Backward	Steam-, Feed-split, TVC, Feed preheater, FTs	BARON, PSO, DE, ABCA, TLBO, GWO, Artificial Electric Field Algorithm	5608.8	8.88

[19]	Backward	Steam-, Feed-split, TVC, Feed preheater, FTs	CONstraint OPTimization, Sine Cosine Algorithm	6008.4	8.45

Present work	Backward	b-MEE	Walrus Optimization Algorithm (WaOA)	7704	5.28
		Steam-, Feed-split, TVC, Feed preheater, FTs (h-MEE)		4705.2	8.84

### 7.3 Implementation of proposed approach

The practical implementation of integrating LFR and wind energy systems with MEE involves careful planning to accommodate site-specific resource availability, equipment installation, and system integration. Operational benefits include enhanced energy efficiency and reliability by combining solar and wind energy, which reduces reliance on conventional fuels and lowers operational costs. Although initial capital investment is significant, the long-term cost savings from reduced energy consumption and potential government incentives make the integration economically advantageous. Environmentally, this approach significantly reduces greenhouse gas emissions and supports sustainability goals by utilizing clean, renewable energy sources. A detailed case study or scenario analysis, such as one focused on an industrial facility in Jodhpur, India, would provide practical insights into the system's effectiveness, highlighting real-world applications, cost implications, and environmental impacts.

### 7.4 Practical implications of linear Fresnel reflectors

The integration of LFR and wind energy significantly enhances system stability by diversifying energy sources, which mitigates the risks associated with dependency on a single energy type. The combination of solar and wind energy provides a more reliable and consistent energy supply for MEE operations. Addressing operational challenges, the system requires advanced control mechanisms to manage the variability of solar and wind resources effectively. Incorporating energy storage solutions can further smooth out these fluctuations. Additionally, this hybrid approach bolsters resilience against energy supply disruptions and price volatility by reducing reliance on conventional energy sources. To sump up, LFR and wind energy with MEE systems can provide benefits in terms of stability, operational efficiency, and overall system robustness.

## 8 Conclusion

The integration of energy-saving schemes (ESSs) with the Walrus optimization algorithm (WaOA) demonstrates significant improvements in the energy efficiency of multiple effect evaporators (MEEs). Through the analysis, it was found that incorporating ESSs led to a remarkable 67.93% increase in the steam economy under nonlinear constraints. Furthermore, including Flash tanks resulted in an additional 5.89% enhancement in the steam economy compared to other ESSs integrated with MEEs, culminating in an overall energy improvement potential of approximately 73%. Comparing findings with existing literature, the results demonstrate competitive performance. Additionally, the hybridization of solar and wind energy sources ensures uninterrupted operation of the MEE despite fluctuations in sunlight availability and wind speeds. This hybrid approach effectively mitigates the heat demand of the MEE, reducing conventional energy utilization by a significant margin of 62% (with linear Fresnel reflectors) and 34% (with wind energy). It is found that the results obtained from the system model are in accordance with the literature. The highlights of the conclusions related to system simulation can be summarized as

• Inclusion of different ESSs presents an effective impact on the design variables especially on the energy efficiency.
• The renewable energy potential for the proposed MEE is very high and hence it can also be helpful for other energy-intensive units or we can also specify the size of the employed renewables.

On account of the fact that the integration of renewables reduces the conventional energy load in various industrial sectors, its use needs to be highly pumped and a forced implementation may be suggested by the government. Given the substantial reduction in conventional energy consumption achieved through renewable integration, there is a compelling case for promoting and mandating the adoption of renewable energy solutions across various industrial sectors. Moreover, investigating the incorporation of other renewable energy sources, such as biomass or geothermal energy, could provide further reductions in conventional energy reliance and improve system efficiency. Scale-up studies would be beneficial to evaluate the practical implementation of these models in various industrial contexts and for different sizes of MEE systems. Additionally, a detailed economic analysis is necessary to understand the cost-effectiveness of integrating ESSs and renewable energy solutions in industrial settings. Lastly, assessing the environmental impact, including potential reductions in greenhouse gas emissions, would offer valuable insights into the broader sustainability benefits of these integrations. Addressing these aspects in future research will contribute to advancing more efficient, cost-effective, and environmentally sustainable industrial processes.

## 9 Acronyms

Table 11 shows the list of the acronyms used in this study.

**Table 11:** Acronyms and their explanation.

Acronym	Full form	Acronym	Full form
*A*	Heat transfer area (m^2^)	*a*	Ambient
*avg*	Average	*Ar*	Rotor area of wind turbine
*c*	Scale parameter of wind speed	*CO*	Condensate out
*cond*	Condensate	*Cp*	Specific heat
*D*	Demand	*CR*	Compression Ratio
*f*	Feed	*DNI*	Direct normal irradiance (W/m^2^)
*i*	Number of variables	*ER*	Expansion Ratio
*in*	Input	*F*	Objective Function
*j*	Outlet	*g*	Equality constraint
*l*	Loss	*h*	Enthalpy (kJ/h)
*L*	Liquor	*H*	Vapor enthalpy (kJ/h)
*m*	Medium	*I*	Radiation (W/m^2^)
*out*	Output	*IAM*	Incidence angle modifier
*r*	Rise	*k*	Feed-split fraction
*v*	Vapor	*L*	Feed flow rate (kg/s)
*s*	Suction	*m*	Vapor fraction is sent to the 7th stage for preheater
*ss*	Steam Separator	*M*	The mass flow rate of water/steam (kg/s)
*n*	Number of stages	*N*	Number of populations
*P*	Power	*BL*	Black Liquor
*Q*	Heat (kW)	*CDF*	Cumulative distribution function
*T*	Temperature (^∘^C)	*CFV*	Condensate Flash Valve
*U*	Overall heat transfer coefficient (kW/m^2^^∘^C)	*CFT*	Condensate Flash Tank
*vs*	Wind speed	*CSP*	Concentrating Solar Power
*V*	Vapor flow rate (kg/s)	*ESS*	Energy Saving Scheme
*x*	Concentration of black liquor	*FFT*	Feed Flash Tank
*y*	Steam-split fraction	*FT*	Flash Tank
*z*	Decision variables	*LFR*	Linear Fresnel Reflector
*MEE*	Multiple Effect Evaporator	*α*	Shape parameter of solar irradiance
*MLT*	Maximum Likelihood Technique	*β*	Scale parameter of solar irradiance
*MMLT*	Modified Maximum Likelihood Technique	*PDF*	Probability Density Function
≓	Latent heat of vaporization (kJ/kg)	*PFT*	Product Flash Tank
*φ*	Shape parameter of wind speed	*SC*	Steam Consumption (kg/s)
*ρ*	Density	*SE*	Steam Economy
*TVC*	Thermo-Vapor Compressor	*WaOA*	Walrus Optimization Algorithm

## Funding

This research is funded by the European University of Atlantic.

## CRediT authorship contribution statement

**Smitarani Pati:** Writing – original draft, Data curation, Conceptualization. **Nandan Kumar Navin:** Writing – original draft, Formal analysis, Conceptualization. **Om Prakash Verma:** Methodology, Formal analysis, Data curation. **Dwesh Kumar Singh:** Software, Project administration, Methodology. **Tarun Kumar Sharma:** Visualization, Software, Resources. **Saurabh Agarwal:** Writing – review & editing, Validation, Formal analysis. **Santos Gracia Villar:** Visualization, Investigation, Funding acquisition. **Luis Alonso Dzul Lopez:** Project administration, Investigation, Data curation. **Imran Ashraf:** Writing – review & editing, Validation, Supervision.

## Declaration of Competing Interest

The authors declare that they have no known competing financial interests or personal relationships that could have appeared to influence the work reported in this paper.

## Data Availability

The data can be requested from the corresponding authors.
